# Viruses exploit growth factor mechanisms to achieve augmented pathogenicity and promote tumorigenesis

**DOI:** 10.1007/s00203-024-03855-2

**Published:** 2024-03-25

**Authors:** Sarvenaz Sabourirad, Evdokia Dimitriadis, Theo Mantamadiotis

**Affiliations:** 1 Department of Obstetrics and Gynaecology, The University of Melbourne, Parkville, VIC Australia; 2 Gynaecology Research Centre, Royal Women’s Hospital, Parkville, VIC Australia; 3 Centre for Reproductive Health, Hudson Institute of Medical Research, Clayton, VIC Australia; 4 Department of Surgery RMH, The University of Melbourne, Parkville, Australia; 5 Department of Microbiology and Immunology, The University of Melbourne, Melbourne, Australia

**Keywords:** Growth factors, Viral exploit, Viral growth factor homologues, Tumorigenesis

## Abstract

Cellular homeostasis is regulated by growth factors (GFs) which orchestrate various cellular processes including proliferation, survival, differentiation, motility, inflammation and angiogenesis. Dysregulation of GFs in microbial infections and malignancies have been reported previously. Viral pathogens exemplify the exploitation of host cell GFs and their signalling pathways contributing to viral entry, virulence, and evasion of anti-viral immune responses. Viruses can also perturb cellular metabolism and the cell cycle by manipulation of GF signaling. In some cases, this disturbance may promote oncogenesis. Viral pathogens can encode viral GF homologues and induce the endogenous biosynthesis of GFs and their corresponding receptors or manipulate their activity to infect the host cells. Close investigation of how viral strategies exploit and regulate GFs, a will shed light on how to improve anti-viral therapy and cancer treatment. In this review, we discuss and provide insights on how various viral pathogens exploit different GFs to promote viral survival and oncogenic transformation, and how this knowledge can be leveraged toward the design of more efficient therapeutics or novel drug delivery systems in the treatment of both viral infections and malignancies.

## Introduction

Growth factors (GF) play a pivotal role in cell proliferation, differentiation, and apoptosis. GFs stimulate intracellular signalling pathways, including the mitogen-activated protein kinase (MAPK), phosphoinositide 3-kinase (PI3K), phospholipase C- gamma, and downstream transcription factors signal transducers and activators of transcription (STATs) or SMAD proteins (Witsch et al. 2010). MAPK signal transduction including classical MAPK (also known as ERK), c-Jun N-terminal kinase/stress-activated protein kinase (JNK/SAPK) and p38 kinase regulate proliferation, differentiation, development, inflammatory responses and apoptosis in mammalian cells (Zhang and Liu 2002).

Viruses leverage various strategies to exploit the components of GF signalling. For instance, viral pathogens may encode GF homologues or GF-like protein domains, thereby hijacking the regulation of GF receptors and promoting viral replication. Several studies have drawn attention to the striking similarities in DNA sequences and protein domains between viral components, host genes and proteins (Johnsson et al. 1986; Waterfield et al. 1983; Yan et al. 2013).

Viruses can manipulate GF receptors and activation of downstream signalling pathways resulting in alterations in expression, dimerization, endocytosis, and recycling of receptors (Lateef and Wise 2018). Many viruses depend on the GF system for host cell entry, replication, and invasion. In some instances, viral manipulation of GFs and downstream pathways disturb cell cycle and metabolism which, may directly or indirectly promote cellular transformation, ultimately contributing to the development and progression of malignancies (Şevik and Sciences 2012b).

Moreover, microbiome imbalance is associated with several pathological conditions, including various metabolic disorders and cancer. The human microbiome comprises various microbes including bacteria, fungi, viruses, which naturally reside in and on our bodies. For example, in human microbiome studies, iridoviridae family DNA sequences were detected in samples which encode viral insulin/IGF-1–like peptides. These viral GF-like peptides may have a key role in various human disorders and diseases (Altindis et al. 2018).

In the context of cancer, the breast tumour microenvironment exhibits an abundance of parapoxvirus genomic sequences, which have the potential to impact the metabolism and proliferation of cancer cells. Breast cancer is a malignancy characterized by significant dysregulation of GFs (Bose et al. 2020).

**Fig. 1:**
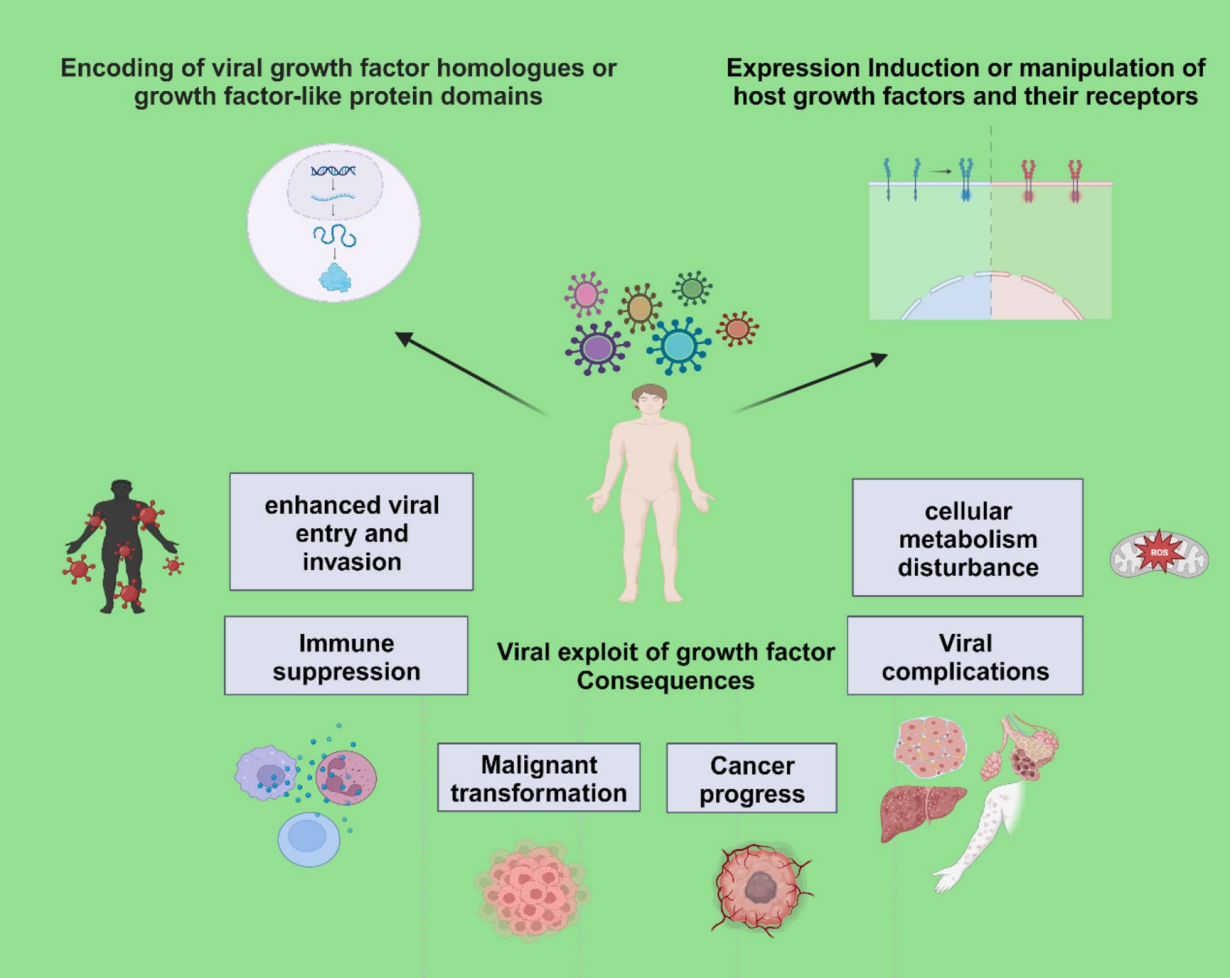
Viral exploitation of growth factors and growth factor-dependent mechanisms and its consequences. Created with BioRender.com

Therefore, investigating the interaction between viruses and GFs is of crucial importance in elucidating the mechanisms that contribute to viral pathogenesis and cancer development. This has the potential to offer valuable insights into how to combat two critical health challenges and pave the way toward innovative therapeutic strategies to improve patient outcome (Fig. 1).

### Viral manipulation of GFs and their signalling pathways

#### Viruses encoding host growth factor homologues/protein domains

Some viruses, such as dsDNA viruses, including poxviruses, herpesviruses, and Iridoviridae, harbor sequences that resemble those found in human cells (Altindis et al. 2018). Through this similarity, these viruses can exploit host genes to mimic endogenous factors leading to the production of growth factor homologues. Subsequently, these GF homologues activate pathways that cater to viral functions, including viral infection, viral metabolic demands and cellular transformation.

A comprehensive bioinformatics driven study revealed that numerous viruses harbor sequences with remarkable similarity to human peptides, including insulin, insulin-like growth factors (IGF)-1 and -2, fibroblast growth factor (FGF) and vascular endothelial growth factor (VEGF). Viral insulin/IGF-1—like peptides (VILPs) are one of the most common factors encoded by the viral family Iridoviridae (Altindis et al. 2018).

Human microbiome studies revealed the presence of DNA sequences belonging to Iridoviridae family in human fecal and blood samples. The VILPs stimulate the autophosphorylation and downstream signalling of IGF-1/insulin receptors and transfer blood glucose to adipocytes and increase the proliferation of fibroblasts (Altindis et al. 2018). Further, Yan et al. (2013) cloned IGF homologue of Singapore grouper iridovirus (SGIV) indicating enhanced SGIV replication and host cell proliferation by promoting enhanced G1/S phase transition (Yan et al. 2013).

Parapoxviruses, abundant in the triple negative breast tumour microenvironment, encode VEGF homologues and modulate the MAPK, ERK and PI3K-AKT pathways. Consequently, these alterations affect cell proliferation and metabolism of both normal and malignant breast cells (Bose et al. 2020). A VEGF homologue encoded by Orf virus, a member of the parapoxvirus, exhibits robust expression during the initial stages of infection, leading to dermal vascular endothelial proliferation and dilatation (Lyttle et al. 1994).

Notably, viral growth factor homologues may differ both among different strains of a virus family and compared to host cellular GFs. These differences are particularly evident in receptor and downstream signalling pathway stimulation. In a study investigating inter-strain variations in amino acid sequence homology, Wise et al. (2003) discovered that parapoxviruses exhibited multiple viral VEGF homologues that functioned similarly in terms of mitogen activity, proliferation of human endothelial cells and angiogenesis. However, these viral VEGF-like proteins exhibited lower affinity for VEGFR-2 compared to the host VEGF. The viral VEGFs exhibited distinct features in terms of VEGFR-2 binding and interaction, vascular permeability and neuropilin-1 binding. By contrast, viral GFs did not bind to either VEGFR-1 nor VEGFR-3 (Wise et al. 2003).

Vaccinia virus (VACV) relies on energy and metabolism of macromolecules to facilitate its multiplication and increases the concentration of intermediates of the tri-carboxylic acid cycle. This is achieved by encoding a viral homologue of cellular epidermal growth factor (EGF) and activation of STAT3 (Pant et al. 2021). Similarly, smallpox growth factor, a protein with an EGF-like domain, can bind to Erb-B1 (EGF receptor), thereby stimulating the growth of primary human keratinocytes and fibroblasts. This phenomenon is exemplified in the skin manifestations associated with infections by this virus (Kim et al. 2004).

An important discovery in 1983, revealed homology between a 90-residue domain of the platelet-derived growth factor (PDGF) and the sequence of p28sis, the putative transforming protein of simian sarcoma virus (SSV). Human fibroblasts transformed by SSV exhibit a similar lifespan and proliferation rate to non-transformed fibroblasts exposed to PDGF (Johnsson et al. 1986; Waterfield et al. 1983).

HIV-1 Tat protein, having sequences that resemble VEGF-A, interacts with vascular endothelial cells and promotes angiogenesis via stimulation of GF receptors (Albini et al. 1996). The HIV-1 Tat protein also shares similar immunosuppressive properties with transforming growth factor beta (TGFβ). Additionally, its 38–62 basic peptide domain displays proliferative effects in chondrocytes, akin to TGFβ actions (Lotz et al. 1994). Moreover, human cytomegaly virus (HCMV) glycoprotein B exhibits GF-like promoting activity by phosphorylating PDGFRα, similar to PDGFRα. This leads to the stimulation of PI3K signalling (Soroceanu et al. 2008).

These observations suggest that viruses encode growth factor homologues or GF-like protein domains to fulfill metabolic needs, facilitate viral replication, and exert mitogenic and transformation effects. Activation of GF pathways by viruses can also promote the supply of nutrients and blood flow to host transformed cells through angiogenesis or alterations in the cell cycle. Viral GF-like homologues exhibit differences, both with cellular GFs and among other viruses (Wise et al. 2003). These variations may lead to potential competition between the GFs and the viral homologues, which can be exploited to combat viral infections, particularly through the synthesis of GF variant-specific antibodies.

#### Viruses induced expression and regulation of host growth factors and receptors

In addition to GF homologue synthesis, diverse viruses have the capacity to induce the expression of host GFs and receptors and are able to manipulate these. An model example of this, is Epstein-Barr virus (EBV). The oncogenic latent membrane protein 1 (LMP1) is a key regulator of EBV oncogenicity which interacts with GFs. In epithelial cells, LMP1 specifically induces EGFR expression by activating a novel form of NF-kB (Kung and Raab-Traub 2010).

In another study, in cell lines expressing LMP1, this protein can stimulate the IGF1R through modified phosphorylation, rather than altering the expression of IGF1R. The mutant forms of LMP1 can enhance the phosphorylation of IGF1R via canonical, NF-kB-dependent mechanisms, accompanied by an increase in mRNA expression and secretion of the IGF1 ligand. Overexpression of IGF has been reported in many studies investigating EBV pathogenesis (Tworkoski and Raab-Traub 2015).

Hepatitis C virus (HCV) is also known to modulate EGF signaling pathways, and is implicated in HCV-mediated oncogenesis. Binding of NS5A non-structural protein of HCV to the growth factor receptor-binding protein 2 (Grb2) adaptor protein inhibits the EGF-induced phosphorylation of ERK1 and ERK2 in infected cells and disrupts the signal transduction pathways. EGF-induced activation of the ERK cascade induces the expression of IFN-dependent genes, but HCV infection may result in their down-regulation (Tan et al. 1999). Additionally, EGFR plays a significant role in HCV entry into human host cells (Lupberger et al. 2011). HCV induces the TGFβ1 expression, contributing to HCV-related hepatic fibrosis (Lin et al. 2010).

Hepatitis B virus (HBV) is also dependent on EGFR endocytosis mechanisms to enter human hepatocytes (Iwamoto et al. 2020). Interestingly, in liver sinusoidal endothelial cells, at high EGF doses, HBV is internalized through clathrin-independent endocytosis, leading to the lysosomal degradation of EGFR and suppression of viral infection. Conversely, at lower EGF concentrations, HBV entry relies on clathrin-mediated endocytosis, resulting in increased viral infection efficiency (Chen et al. 2020). Similar to HCV, in initial infection stages, the HBV X antigen trans-activates the TGFβ1 promoter. TGFβ1 overexpression is prevalent in liver diseases and significantly impacts HBV pathogenesis, including hepatocellular carcinoma (Yoo et al. 1996).

EGFR also interacts with the main viral envelope glycoprotein of human cytomegalovirus (HCMV), gB, to internalise this pathogen (Wang et al. 2003). HCMV selectively targets different cell types based on its two virion glycoprotein complexes. The trimeric complex primarily triggers fibroblasts, whereas the pentameric form infects epithelial and endothelial cells. HCMV exploits various GF receptors to establish infection within host cells. Genome-wide CRISPR screens identified the extracellular domain of platelet-derived growth factor α (PDGFRα) as one of the crucial components for infection by HCMV, containing only trimeric complex (Wu et al. 2018). In placental tissue, PDGFRα was the important form for virions lacking the pentameric complex (Naing et al. 2020). Soluble derivatives of PDGFRα can inhibit HCMV infection in various cell types by binding to and neutralizing cell-free virus particles (Stegmann et al. 2017).

Other viral pathogens including human papillomavirus type 16 (HPV16), influenza A virus (IAV) and zika virus exploits EGFR and its downstream signaling pathways for viral entry (Bannach et al. 2020; Lai et al. 2020; Sabino et al. 2021). Moreover, internalisation of filoviruses, such as Ebola and Marburg, may be blocked by inhibitors of EGFR, tyrosine protein kinase Met (c-Met), and the insulin receptor (InsR)/IGF1R (Stewart et al. 2021).

In SARS-CoV-2 infection, EGFR or PDGFR are reported to be the most abundant GFs exploited for the regulation of virus replication. The inhibition of GF downstream signalling can prevent virus replication and fibrosis in patients with idiopathic pulmonary fibrosis (Klann et al. 2020). Moreover, some complications in SARS-CoV-2 infected patients, such as coagulopathy, have been linked to the dysregulated expression of several GFs, including VEGF, PDGF, IGF-1 and TGF-β but the exact mechanism by which the GFs exert these co-pathologies remain elusive (Ahmad et al. 2022). A strong association was also observed between PDGF and FGF-2 expression and disease severity (Petrey et al. 2021).

Respiratory syncytial virus (RSV) infection, commonly infecting children under 2 years, upregulates the expression of nerve growth factor (NGF) and its receptor. NGF causes neurogenic inflammation in RSV-infected airways by upregulation of substance P and neurokinin 1 receptor (Hu et al. 2002). Viral strategies to exploit GFs are summarised in Table 1.

**Table 1:** Viruses exploit growth factors and their pathways

Virus type	Exploited growth factor	Function	References
Epstein-Barr virus (EBV)	Induction of EGFR expression, stimulation and expression of IGF1R and IGF1 ligand	Oncogenesis and pathogenesis	Kung and Raab-Traub (2010), Tworkoski and Raab-Traub (2015)
Hepatitis C virus (HCV)	Modulation of EGF signalling pathways, EGFR regulation, TGFβ and VEGF overexpression	Oncogenesis, downregulation of IFN-dependent genes, viral entry, angiogenesis	Lin et al. (2010), Lupberger et al. (2011), Tan et al. (1999), Nasimuzzaman et al. (2007)
Hepatitis B virus (HBV)	EGFR regulation, TGFβ overexpression	Viral entry, pathogenesis, and oncogenesis	Chen et al. (2020), Iwamoto et al. (2020), Yoo et al. (1996)
Human papillomavirus type 16 (HPV16)	EGF and EGFR regulation	Viral entry, oncogenesis	Bannach et al. (2020)
Human cytomegalovirus (HCMV)	EGFR, PDGFRα, PDGF-D regulation, encoding of viral PDGF homologue	Viral entry, phosphorylation of PDGFRα and stimulation of PI3K signalling, angiogenesis	Naing et al. (2020), Wang et al. (2003), Wu et al. (2018), Soroceanu et al. (2008), Krenzlin et al. (2019)
Influenza A virus (IAV)	EGFR regulation, activation of EGFR	Viral entry, inhibiting of IFNγ production and suppression of antiviral immune response	Bannach et al. (2020), Lai et al. (2020)
Zika virus	EGFR regulation	Viral entry	Sabino et al. (2021)
Filoviruses	EGFR, (c-Met), and InsR/ IGF1R regulation	Viral entry	Stewart et al. (2021)
SARS-CoV-2	EGFR or PDGFR regulation	Regulation of virus replication	Klann et al. (2020)
Respiratory syncytial virus (RSV)	Upregulation of NGF and its receptor	Increase of inflammation and pathogenicity	Hu et al. (2002)
Iridoviridae	Encoding of viral Insulin/IGF homologue	Stimulation of signalling of IGF-1/insulin receptors, blood glucose uptake, enhanced the virus replication and host cell proliferation	Altindis et al. (2018), Yan et al. (2013)
Parapoxvirus	Encoding of viral VEGF homologue	Metabolism and proliferation alterations in host cells, angiogenesis	Bose et al. (2020), Lyttle et al. (1994)
Simian sarcoma virus	Encoding of viral PDGF homologue	Proliferation increase in host cells	Johnsson et al. (1986), Waterfield et al. (1983)
Smallpox virus	Encoding of viral EGF homologue	Stimulation of EGFR and proliferation increase in host cells	Kim et al. (2004)
Vaccinia virus	Encoding of viral EGF homologue	Metabolism and proliferation increase in host cells, STAT3 activation	Pant et al. (2021)
Human immunodeficiency virus (HIV)	Encoding of viral VEGF and TGFβ homologue	Proliferation of host and angiogenesis, immune suppression	Lotz et al. (1994)
Rhinovirus (RV)	Activation of EGFR	Inhibiting of IFNγ production and suppression of antiviral immune response	Ueki et al. (2013)
Kaposi’s sarcoma-associated herpesvirus (KSHV/HHV8)	Regulation of VEGF	Oncogenesis, angiogenesis	Bais et al. (1998)
Human T-lymphotropic virus 1(HTLV-I)	Leading to a truncated PDGFβ-receptor	Transforming capability in T cells	Chi et al. (1997)

## Immunomodulatory effect of growth factors in viral infection

Recent studies provide compelling evidence that viruses utilise GFs to evade host immune responses. Disruption of anti-viral immune responses can arise from the direct immunoinhibitory effects of GFs. TGFβ is a key factor with broad inhibitory effects on immune cells, which can be upregulated with certain viruses such as HBV, HIV-1 and HCV.

TGFβ can suppress IFN responses associated with rhinovirus (RV) infection, thereby increasing the likelihood of asthma and RV replication (Bedke et al. 2012). Natural killer (NK) cells are considered as one of the most potent immune cells in the eradication of viral infection. However, TGFβ can suppress NK cell activation (Wilson et al. 2011). Similarly, CD8+ T cell responses in chronic viral infections are influenced by TGFβ-Smad signalling by inhibiting TGFβ in T cells which can eliminate viral infections. TGFβ induces apoptosis in virus-specific CD8+ T cells by upregulating the proapoptotic protein, Bim (Tinoco et al. 2009).

In addition to cellular immunity, TGFβ1 can disrupt humoral immunity in viral diseases. PBMCs derived from HIV patients release higher levels of TGFβ1 and exhibit abnormal cellular and humoral immune responses. B cells from HIV patients exhibit lower proliferative capacity and release of immunoglobulins (Kekow et al. 1991).

IGFs inhibit the maturation of dendritic cells (DCs), antigen presentation, and activation of antigen-specific CD8+ T cells. These effects are mediated via reduced ERK1/2 phosphorylation and p38 dephosphorylation. DCs play a crucial role in NK cell stimulation against viral diseases (Huang et al. 2015). The differentiation and proliferation of DCs is also inhibited by VEGF, extensively reviewed previously (Kusmartsev et al. 2006).

The immunosuppressive effects of IGF-1 on anti-viral immunity are also mediated through enhanced proliferation of regulatory T (Treg) cells. This finding is corroborated by the observation that ablation of the IGF-1 receptor on Treg cells disrupts the proliferation of these cells (Bilbao et al. 2014). Moreover, IGF-1 inhibits Th1- and NK mediated cellular immune responses via stimulation of IL-10 production in T cells (Kooijman and Coppens 2004). As a result, viruses inducing IGF expression can adversely affect anti-viral immune response.

GF-dependent modulation of the immune system has been extensively documented in respiratory viruses such as influenza virus and RV. This modulation occurs through the activation of EGFR, which hinders the production of IFNγ and suppresses the antiviral immune response via the IFN regulatory factor (IRF)-1 pathway (Ueki et al. 2013).

Similarly, HCV can manipulate the EGF pathways by binding to Grb2 and inhibiting EGF-induced phosphorylation of ERK. Since the activation of the ERK cascade by EGF is crucial for the induction of IFN gene expression, it is proposed that HCV may down-regulate the expression of IFN genes (Tan et al. 1999). Moreover, stimulation of STAT3 via EGFR and MAPK signaling pathways may attenuate the antiviral effects of type I IFN (Pant et al. 2021; Wang et al. 2011).

Additionally, fibroblast growth factor (FGF)-related interference of IFN signalling facilitates viral replication. FGFs exhibit the ability to promote the replication of various viruses, including HSV-1, lymphocytic choriomeningitis virus, and Zika virus. FGFs exert their inhibitory effect on IFN-stimulated genes (ISGs) at the transcriptional level, requiring the activity of FGF receptor kinase and proteasomes (Maddaluno et al. 2020).

In summary, the aforementioned findings highlight the ability of GFs to facilitate viral infections directly or indirectly. The GFs can dampen immune responses against viruses, particularly by interfering with IFNγ response and suppressing immune cells.

## Virus-growth factor interactions in cancer development and progression

Normal, non-transformed cells rely on specific GFs for their proliferation. Some GFs selectively stimulate specific cell types, while others, like EGF, have a broad effect on various cell types, including both epithelial and mesenchymal cells. Different growth factors synergistically enhance each other’s mitogenic activity, exerting their effects at various stages of the cell cycle (Grassmann and Fleckenstein 2006).

GFs have the potential to support tumour progression, clonal expansion, metastasis, angiogenesis, and establishment of distant niches, as well as drug resistance (Witsch et al. 2010). In line with this, EGFR is overexpressed in many types of cancer and EGFR antagonists are being evaluated in cancer therapy with promising results. These antagonists, in addition to direct effects, reinforce the immune system by disrupting the action of Treg cells (MacDonald and Zaiss 2017).

Viral pathogens can induce cellular transformation and promote the extensive proliferation and progression of cancer cells by exploiting GF functions. This can be achieved through viral oncogenes or mutations that facilitate the progression of the cancer cell niche. Several viruses, including EBV, human herpesvirus (HHV) 8, HPV- 16 and 18, HBV, HCV virus and retroviruses (RV). RV are classified as oncogenic viruses and exhibit causative roles in the induction of proliferation of benign or malignant infected cells. These viral pathogens can lead to tumour development by viral oncogenes which impact GF function (Şevik and Sciences 2012a).

HPV 16 and 18 are primarily associated with cervical carcinoma. These pathogens can immortalize normal human mammary epithelial cells by integrating into the host DNA, expressing HPV genes and producing the HPV E7 protein (Band et al. 1990). Alternative splicing of the HPV16 E6/E7 ORF cassette is regulated by the EGF pathway. This splicing process is responsible for producing viral transforming proteins such as E6 and E7 (Rosenberger et al. 2010).

Kaposi’s sarcoma (KSHV)-associated HHV8 (KSHV/HHV8) carries oncogenic proteins that play a role in sarcoma pathogenesis. Specifically, the KSHV G-protein-coupled receptor is a key factor in cell transformation, tumorigenicity and angiogenesis, mediated by factors such as VEGF and Kaposi’s spindle-cell growth factor (Bais et al. 1998).

Human T lymphotropic virus 1(HTLV-I) can integrate an undeleted HTLV-I provirus into the PDGFβ-receptor gene resulting in a truncated PDGFβ-receptor with transforming capability in T cells (Chi et al. 1997). In SSV, the putative transforming protein exhibits similarity to PDGF (Johnsson et al. 1986; Waterfield et al. 1983).

Another example of an oncogenic viral protein is EBV-related LMP1, which facilitates the binding and interaction of EGFR and STAT3 with the cyclin D1 promoter. EBV-related LMP1 enhances the activity of the cyclin D1 promoter (Xu et al. 2013). In epithelial cells, LMP1 induces the expression of the EGFR (Kung and Raab-Traub 2010). In the case of simian virus 40 (SV40), the large T-antigen can transform various cell types via the IGF-I receptor (Sell et al. 1993).

In addition to carrying oncogenes dealing with GFs, viruses can promote cancer progression by triggering the host GF pathways. As discussed earlier, viral exploitation of GFs can contribute to cancer progression. This is achieved by immune suppression, disruption of the cell cycle and metabolism, angiogenesis promotion and fibrosis induction. Studies on the microbiome have revealed viral sequences within the tumour microenvironment, potentially influencing both the progression of cancer and the outcomes of treatment (Cantalupo et al. 2018).

TGFβ plays a vital role to progress tumours by immune suppression. HBV and HCV can increase TGFβ expression in tumour cells (Lin et al. 2010; Yoo et al. 1996). HCMV can promote tumor angiogenesis leading to reduced survival in HCMV^+^ mouse models. Specifically, the virus exploits PDGF-D for pericyte recruitment, angiogenesis and tumour growth. These effects are inhibited by the antiviral drug, Cidofovir (Krenzlin et al. 2019).

HCV also plays a significant role in hepatic neoangiogenesis by enhancing the expression of TGFβ2 and VEGF, activating endothelial cells and increasing CD34 expression. This virus activates signaling pathways such as JNK, p38 and ERK, considered the major pathways involved in the regulation of TGFβ2 and VEGF proteins (Hassan et al. 2009). The core protein of HCV upregulates the expression of TGFβ1 by activating the TGFβ1 promoter. Disruption of the MAPK pathway inhibits TGFβ1 upregulation by this viral protein (Taniguchi et al. 2004). HCV stabilizes HIF-1α through oxidative stress and calcium signaling. Multiple pathways, including NF-kB, STAT-3, PI3-K/Akt, and p42/44 MAPK, contribute to this stabilization, leading to the expression and release of VEGF (Nasimuzzaman et al. 2007).

To summarize, there are various mechanisms through which viruses support tumor cells. The GF-related viral strategies outlined here contribute to tumor progression and invasion. These interactions within the virus-GF system promote malignant cell proliferation, angiogenesis, tumor invasion and immune suppression, thereby facilitating virus persistence.

## Growth factor-virus interaction for gene and drug delivery

Given their role in infection, diverse types of viral pathogens have been recruited in the pharmaceutical field, with the aim of drug and gene delivery. Retroviruses, HSVs and adenoviruses are employed for clinical application given their high infection efficiency (Mesri et al. 1995; Nayerossadat et al. 2012). Moreover, the viral components can be used to modify other non-viral vectors, including diverse polymeric drug carriers. HIV Tat protein can carry proteins with a molecular weight of more than 100 kDa, 40 nm particles and 200 nm liposomes into target cells and act as a cell entry peptide (Seelig et al. 2011).

Many studies have emphasized a role for GFs in virus internalization, so the investigation of GF-virus interactions for drug and gene delivery may open new avenues for novel drug delivery. GF-virus interaction is exemplified by the importance of EGFR as a co-receptor for adeno-associated virus serotype 6 (AAV6) to transduce the target cells. AAV6 is dependent on EGFR to efficiently function as a vector to deliver drugs to target cells (Weller et al. 2010).

Moreover, McCart and colleagues (2001) designed a vaccinia virus vector by deleting several genes. Deletion of thymidine kinase (TK) and viral GF improved the specificity of this viral vector for use in cancer therapy. Specifically, the viral GF acts as a mitogen which stimulates surrounding cells for vaccinia infection. Genetic deletion of this viral GF reduced viral replication in resting cells. In the absence of vaccinia GF, the division rate of host cells decreases. This phenomenon improves tumour targeting and at the same time decreases viral pathogenicity. As a result, this TK-and VGF-mutant vaccinia virus vector exhibits high replication in tumour cells with significant oncolytic effects in vivo (McCart et al. 2001).

Additionally, GF signalling improves viral vector delivery by increasing the proliferation of host target cells. For example, for murine leukemia virus (MLV) to work efficiently as a therapeutic vector, actively dividing cells are required. However, due to the low rate of central nervous system (CNS) cellular division, this vector is unsuitable for applications targeting the adult CNS. However, basic FGFs trigger cell proliferation in adult brain and significantly foster MLV-based gene transfer (King et al. 2000).

In some cases, viral vectors are used to carry GF genes. This has been reported in the case of VEGF during myocardial ischemia. Mesri et al. (1995) designed a replication-defective HSV-1 amplicon vector which carried the human VEGF-165 cDNA under the transcriptional control of the HSV immediate-early 4/5 promoter. This vector exhibited a remarkable angiogenic response in vivo (Mesri et al. 1995). Similarly, the exogenous expression of VEGF by AAVH9-VEGF gene transfer, offered neuroprotection in ischemic brain injury (Shen et al. 2006). Moreover, replication-defective HSV vectors expressing NGF showed promising results with a significant increase of NGF in the bladder in a rat model of diabetic cystopathy (Sasaki et al. 2004).

Another aspect of GF-virus interactions are their applications in oncolytic virus (OVs) therapy which is a promising cancer treatment. OVs are engineered viruses which are virulence free and do not infect healthy cells. These modified viruses can utilise GF receptors to transfect tumour cells with upregulated GF receptors. Several variations of oncolytic HSV-1 has been designed to target human epidermal growth factor receptor (HER) 2-positive breast cancer cells. This oncolytic virus can target the EGFR to also transfect glioblastoma cells (Jamieson et al. 2020).

These studies highlight that viral vector-GF signalling interactions possess the potential for efficient drug and gene delivery. These vectors can be engineered with the aim of removing infection risk and ability for cell-specific targeted delivery. Since GF receptors are highly expressed in tumour cells, the viral vectors interacting with the GF system could have important implications for efficient cancer drug delivery. Additionally, the viral vector interaction with the GF network has also provided promise in regenerative medicine as viral vectors are commonly used to transfer GF genes into human tissues to prevent the degenerative diseases, including brain and heart ischemia, as well as diabetes.

## Concluding remarks

This review highlights that viral pathogens utilise host GF pathways to survive and replicate. GF manipulation by viral pathogens may lead to several consequences including cellular transformation, tumour formation and support, increased viral replication and altered cellular metabolism, and viral complications. GR receptors can be considered as critical pathways to internalise viral pathogens, indicated in many viral infections, including infections by HCV, HCMV, HPV, EBV and filoviruses. Notably, one of the most mutated and/or overexpressed GF receptor in cancer, the EGFR, is also one of the most abundantly receptors co-opted by viruses, for their transfection into host cells.

Viral pathogens leverage GF signaling pathways through viral GF homologue synthesis and GF-like protein domains, and host GF expression and co-option. Viral pathogens can lead to overexpression of immune suppressing GFs. Viruses can exploit host GF pathways and alter the expression, activation and normal function of GF receptors for oncogenic features, viral entry and distribution and immune suppression. IFNγ signalling mediates the immune system disruption by viral pathogens. IFNs can stimulate both the innate and adaptive immune responses. Disruption of IFNγ is also important in cancer progression by contributing to immune system paralysis.

As discussed above, viral pathogens may manipulate GF pathways and lead to malignancy development and progress through either oncogenes or supporting tumour cells. Viral oncogenes mainly trigger the EGF and PDGF pathways. Tumour development is achieved by promoting angiogenesis, modifying cell cycle, metabolism, and immune suppression. Additionally, virus-stimulated GF overexpression is sometimes accompanied by pathological consequences including fibrosis which contributes to oncogenesis. Indeed, even in some non-viral tumours, the tumour microenvironment has been reported to harbor viral microbiome components that can regulate cancer cell proliferation and metabolism, potentially hindering the effectiveness of treatments (Bose et al. 2020).

**Fig. 2:**
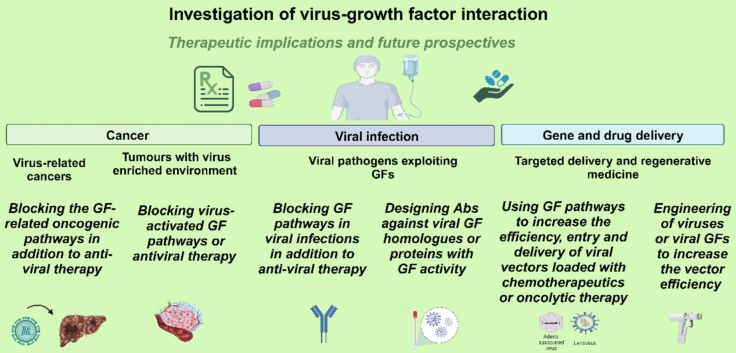
Proposed treatments leveraging growth factor and viral pathogen interaction. Created with www.BioRender.com

Altogether, the insights gained from many studies have the potential to guide the prevention and treatment of viral infection and cancer. For example, the differences among viral GF homologues and host GFs can be investigated more closely to design specific antibodies which are able to compete with these viral products to inhibit or block various viral functions. Moreover, non-infection viral vectors can be designed to take advantage of upregulated GF pathways for targeted drug and gene delivery to improve cancer therapy, shown in Fig. 2. Continued efforts are needed to define viral proteins with GF activity given their importance in cancer biology. A deeper understanding of how GFs impact the immune system will enhance the understanding of the tumour microenvironment, especially in terms of cancers enriched in GF mutations.

## Data Availability

The data obtained for the conduction of this research project are available through the references listed.

## Abbreviations

GF: Growth factor
IGF: Insulin-like growth factors
FGF: Fibroblast growth factor
VEGF: Vascular endothelial growth factor
EGF: Epidermal growth factor
PDGF: Platelet-derived growth factor
VACV: Vaccinia virus
SSV: Simian sarcoma virus
TGFβ: Transforming growth factor beta
HBV: Hepatitis B virus
HCV: Hepatitis C virus
EBV: Epstein-Barr virus
HPV: Human papillomavirus
LMP1: Latent membrane protein 1
HCMV: Human cytomegalovirus
HHV: Human herpesvirus
NGF: Nerve growth factor
MAPK: Mitogen-activated protein kinase
PI3K: Phosphoinositide 3-kinase
OVs: Oncolytic viruses

## References

[CR1] Ahmad F, Kannan M, Ansari AWJC, Reviews GF (2022). Role of SARS-CoV-2-induced cytokines and growth factors in coagulopathy and thromboembolism. Cytokine Growth Fact Rev.

[CR2] Albini A, Soldi R, Giunciuclio D, Giraudo E, Benelli R, Primo L, Noonan D, Salio M, Camussi G, Rock WJNm,  (1996). The angiogenesis induced by HIV–1 Tat protein is mediated by the Flk–1/KDR receptor on vascular endothelial cells. Nat Med.

[CR3] Altindis E, Cai W, Sakaguchi M, Zhang F, GuoXiao W, Liu F, De Meyts P, Gelfanov V, Pan H, DiMarchi RJ (2018). Viral insulin-like peptides activate human insulin and IGF-1 receptor signaling: a paradigm shift for host–microbe interactions. Proc Natl Acad Sci.

[CR4] Bais C, Santomasso B, Coso O, Arvanitakis L, Raaka EG, Gutkind JS, Asch AS, Cesarman E, Gerhengorn MC, Mesri EAJN (1998). G-protein-coupled receptor of Kaposi’s sarcoma-associated herpesvirus is a viral oncogene and angiogenesis activator. Nature.

[CR5] Band V, Zajchowski D, Kulesa V, Sager RJ (1990). Human papilloma virus DNAs immortalize normal human mammary epithelial cells and reduce their growth factor requirements. Proc Natl Acad Sci.

[CR6] Bannach C, Brinkert P, Kühling L, Greune L, Schmidt MA, Schelhaas MJ (2020). Epidermal growth factor receptor and Abl2 kinase regulate distinct steps of human papillomavirus 16 endocytosis. J Virol.

[CR7] Bedke N, Sammut D, Green B, Kehagia V, Dennison P, Jenkins G, Tatler A, Howarth PH, Holgate ST, Davies DE (2012). Transforming growth factor-beta promotes rhinovirus replication in bronchial epithelial cells by suppressing the innate immune response. PLoS ONE.

[CR8] Bilbao D, Luciani L, Johannesson B, Piszczek A, Rosenthal N (2014). Insulin-like growth factor-1 stimulates regulatory T cells and suppresses autoimmune disease. EMBO Mol Med.

[CR9] Bose D, Banerjee S, Singh RK, Wise LM, Robertson ES (2020). Vascular endothelial growth factor encoded by Parapoxviruses can regulate metabolism and survival of triple negative breast cancer cells. Cell Death.

[CR10] Cantalupo PG, Katz JP, Pipas JM (2018). Viral sequences in human cancer. Virology.

[CR11] Chen S-W, Himeno M, Koui Y, Sugiyama M, Nishitsuji H, Mizokami M, Shimotohno K, Miyajima A, Kido TJ (2020). Modulation of hepatitis B virus infection by epidermal growth factor secreted from liver sinusoidal endothelial cells. Sci Rep.

[CR12] Chi KD, McPhee RA, Wagner AS, Dietz JJ, Pantazis P, Goustin ASJO (1997). Integration of proviral DNA into the PDGF β-receptor gene in HTLV-I-infected T cells results in a novel tyrosine kinase product with transforming activity. Oncogene.

[CR13] Grassmann R, Fleckenstein B (2006). Viral oncogenesis. Encyclopedic reference of genomics and proteomics in molecular medicine.

[CR14] Hassan M, Selimovic D, Ghozlan H, Abdel-kader OJH (2009). Hepatitis C virus core protein triggers hepatic angiogenesis by a mechanism including multiple pathways. Hepatology.

[CR15] Hu C, Wedde-Beer K, Auais A, Rodriguez MM, Piedimonte G (2002). Nerve growth factor and nerve growth factor receptors in respiratory syncytial virus-infected lungs. Am J Physiol-Lung Cell Mol Physiol.

[CR16] Huang C-T, Chang M-C, Chen Y-L, Chen T-C, Chen C-A, Cheng W-FJCl,  (2015). Insulin-like growth factors inhibit dendritic cell-mediated anti-tumor immunity through regulating ERK1/2 phosphorylation and p38 dephosphorylation. Cancer Lett.

[CR17] Iwamoto M, Saso W, Nishioka K, Ohashi H, Sugiyama R, Ryo A, Ohki M, Yun J-H, Park S-Y, Ohshima TJ (2020). The machinery for endocytosis of epidermal growth factor receptor coordinates the transport of incoming hepatitis B virus to the endosomal network. J Biol Chem.

[CR18] Jamieson TR, Poutou J, Ilkow CS (2020). Redirecting oncolytic viruses: engineering opportunists to take control of the tumour microenvironment. Cytokine Growth Factor Rev.

[CR19] Johnsson A, Betsholtz C, Heldin C-H, Westermark BJ (1986). The phenotypic characteristics of simian sarcoma virus-transformed human fibroblasts suggest that the v-sis gene product acts solely as a PDGF receptor agonist in cell transformation. EMBO J.

[CR20] Kekow J, Wachsman W, McCutchan JA, Gross W, Zachariah M, Carson D, Lotz MJ (1991). Transforming growth factor-beta and suppression of humoral immune responses in HIV infection. J Clin Investig.

[CR21] Kim M, Yang H, Kim S-K, Reche PA, Tirabassi RS, Hussey RE, Chishti Y, Rheinwald JG, Morehead TJ, Zech TJ (2004). Biochemical and functional analysis of smallpox growth factor (SPGF) and anti-SPGF monoclonal antibodies. J Biol Chem.

[CR22] King L, Mitrophanous K, Clark L, Kim V, Rohll J, Kingsman A, Colello RJ (2000). Growth factor enhanced retroviral gene transfer to the adult central nervous system. Gene Ther.

[CR23] Klann K, Bojkova D, Tascher G, Ciesek S, Münch C, Cinatl J (2020). Growth factor receptor signaling inhibition prevents SARS-CoV-2 replication. Mol Cell.

[CR24] Kooijman R, Coppens AJ (2004). Insulin-like growth factor-I stimulates IL-10 production in human T cells. J Leukocyte Biol.

[CR25] Krenzlin H, Behera P, Lorenz V, Passaro C, Zdioruk M, Nowicki MO, Grauwet K, Zhang H, Skubal M, Ito HJ (2019). Cytomegalovirus promotes murine glioblastoma growth via pericyte recruitment and angiogenesis. J Clin Investig.

[CR26] Kung C-P, Raab-Traub NJ (2010). Epstein-Barr virus latent membrane protein 1 modulates distinctive NF-κB pathways through C-terminus-activating region 1 to regulate epidermal growth factor receptor expression. J Virol.

[CR27] Kusmartsev S, Gabrilovich DI (2006). Effect of tumor-derived cytokines and growth factors on differentiation and immune suppressive features of myeloid cells in cancer. Cancer Metastasis Rev.

[CR28] Lai KM, Goh BH, Lee WL (2020). Attenuating influenza a virus infection by heparin binding EGF-like growth factor. Growth Factors (chur, Switzerland).

[CR29] Lateef Z, Wise LM (2018). Exploitation of receptor tyrosine kinases by viral-encoded growth factors. Growth Factors.

[CR30] Lin W, Tsai WL, Shao RX, Wu G, Peng LF, Barlow LL, Chung WJ, Zhang L, Zhao H, Jang JY (2010). Hepatitis C virus regulates transforming growth factor β1 production through the generation of reactive oxygen species in a nuclear factor κB–dependent manner. Gastroenterology.

[CR31] Lotz M, Clark-Lewis I, Ganu VJ (1994). HIV-1 transactivator protein Tat induces proliferation and TGF beta expression in human articular chondrocytes. J Cell Biol.

[CR32] Lupberger J, Zeisel MB, Xiao F, Thumann C, Fofana I, Zona L, Davis C, Mee CJ, Turek M, Gorke SJ (2011). EGFR and EphA2 are host factors for hepatitis C virus entry and possible targets for antiviral therapy. Nat Med.

[CR33] Lyttle DJ, Fraser KM, Fleming SB, Mercer AA, Robinson AJ (1994). Homologs of vascular endothelial growth factor are encoded by the poxvirus orf virus. J Virol.

[CR34] MacDonald F, Zaiss DM (2017). The immune system’s contribution to the clinical efficacy of EGFR antagonist treatment. Front Pharmacol.

[CR35] Maddaluno L, Urwyler C, Rauschendorfer T, Meyer M, Stefanova D, Spörri R, Wietecha M, Ferrarese L, Stoycheva D, Bender DJ (2020). Antagonism of interferon signaling by fibroblast growth factors promotes viral replication. EMBO Mol Med.

[CR36] McCart JA, Ward JM, Lee J, Hu Y, Alexander HR, Libutti SK, Moss B, Bartlett DL (2001). Systemic cancer therapy with a tumor-selective vaccinia virus mutant lacking thymidine kinase and vaccinia growth factor genes. Cancer Res.

[CR37] Mesri EA, Federoff HJ, Brownlee MJ (1995). Expression of vascular endothelial growth factor from a defective herpes simplex virus type 1 amplicon vector induces angiogenesis in mice. Circul Res.

[CR38] Naing Z, Hamilton ST, van Zuylen WJ, Scott GM, Rawlinson WD (2020). Differential expression of PDGF receptor-α in human placental trophoblasts leads to different entry pathways by human cytomegalovirus strains. Sci Reports.

[CR39] Nasimuzzaman M, Waris G, Mikolon D, Stupack DG, Siddiqui AJ (2007). Hepatitis C virus stabilizes hypoxia-inducible factor 1α and stimulates the synthesis of vascular endothelial growth factor. J Virol.

[CR40] Nayerossadat N, Maedeh T, Ali P (2012). Viral and nonviral delivery systems for gene delivery. Adv Biomed Res.

[CR41] Pant A, Dsouza L, Cao S, Peng C, Yang ZJ (2021). Viral growth factor-and STAT3 signaling-dependent elevation of the TCA cycle intermediate levels during vaccinia virus infection. PLoS Pathog.

[CR42] Petrey AC, Qeadan F, Middleton EA, Pinchuk IV, Campbell RA, Beswick EJ (2021). Cytokine release syndrome in COVID-19: innate immune, vascular, and platelet pathogenic factors differ in severity of disease and sex. J Leucocyte Biol.

[CR43] Rosenberger S, Arce JD-C, Langbein L, Steenbergen RD, Rösl FJ (2010). Alternative splicing of human papillomavirus type-16 E6/E6* early mRNA is coupled to EGF signaling via Erk1/2 activation. Proc Natl Acad Sci.

[CR44] Sabino C, Bender D, Herrlein ML, Hildt E (2021). The epidermal growth factor receptor is a relevant host factor in the early stages of the zika virus life cycle in vitro. Virology.

[CR45] Sasaki K, Chancellor MB, Goins WF, Phelan MW, Glorioso JC, De Groat WC, Yoshimura NJD (2004). Gene therapy using replication-defective herpes simplex virus vectors expressing nerve growth factor in a rat model of diabetic cystopathy. Diabetes.

[CR46] Seelig J, Ziegler A, Klocek GJ (2011). Cell penetrating peptides. How do they cross membranes?. Biophys J.

[CR47] Sell C, Rubini M, Rubin R, Liu J-P, Efstratiadis A, Baserga RJ (1993). Simian virus 40 large tumor antigen is unable to transform mouse embryonic fibroblasts lacking type 1 insulin-like growth factor receptor. Proc Natl Acad Sci.

[CR48] Şevik M (2012). Oncogenic viruses and mechanisms of oncogenesis. Turk J Vet Anim Sci.

[CR49] Şevik M (2012). Oncogenic viruses and mechanisms of oncogenesis. Turk J Vet.

[CR50] Shen F, Su H, Fan Y, Chen Y, Zhu Y, Liu W, Young WL, Yang G-Y (2006). Adeno-associated viral vector-mediated hypoxia-inducible vascular endothelial growth factor gene expression attenuates ischemic brain injury after focal cerebral ischemia in mice. Stroke.

[CR51] Soroceanu L, Akhavan A, Cobbs CS (2008). Platelet-derived growth factor-α receptor activation is required for human cytomegalovirus infection. Nature.

[CR52] Stegmann C, Hochdorfer D, Lieber D, Subramanian N, Stöhr D, Laib Sampaio K, Sinzger CJ (2017). A derivative of platelet-derived growth factor receptor alpha binds to the trimer of human cytomegalovirus and inhibits entry into fibroblasts and endothelial cells. PLoS Pathog.

[CR53] Stewart CM, Phan A, Bo Y, LeBlond ND, Smith TK, Laroche G, Giguère PM, Fullerton MD, Pelchat M, Kobasa DJ (2021). Ebola virus triggers receptor tyrosine kinase-dependent signaling to promote the delivery of viral particles to entry-conducive intracellular compartments. PLoS Pathog.

[CR54] Tan S-L, Nakao H, He Y, Vijaysri S, Neddermann P, Jacobs BL, Mayer BJ, Katze MG (1999). NS5A, a nonstructural protein of hepatitis C virus, binds growth factor receptor-bound protein 2 adaptor protein in a Src homology 3 domain/ligand-dependent manner and perturbs mitogenic signaling. Proc Natl Acad Sci.

[CR55] Taniguchi H, Kato N, Otsuka M, Goto T, Yoshida H, Shiratori Y, Omata MJ (2004). Hepatitis C virus core protein upregulates transforming growth factor-β1 transcription. J Med Virol.

[CR56] Tinoco R, Alcalde V, Yang Y, Sauer K, Zuniga EIJI (2009). Cell-intrinsic transforming growth factor-β signaling mediates virus-specific CD8+ T cell deletion and viral persistence in vivo. Immunity.

[CR57] Tworkoski K, Raab-Traub NJ (2015). LMP1 promotes expression of insulin-like growth factor 1 (IGF1) to selectively activate IGF1 receptor and drive cell proliferation. J Virol.

[CR58] Ueki IF, Min-Oo G, Kalinowski A, Ballon-Landa E, Lanier LL, Nadel JA, Koff JL (2013). Respiratory virus–induced EGFR activation suppresses IRF1-dependent interferon λ and antiviral defense in airway epithelium. J Exp Med.

[CR59] Wang X, Huong S-M, Chiu ML, Raab-Traub N, Huang E-SJ (2003). Epidermal growth factor receptor is a cellular receptor for human cytomegalovirus. Nature.

[CR60] Wang W-B, Levy DE, Lee C-KJ (2011). STAT3 negatively regulates type I IFN-mediated antiviral response. J Immunol.

[CR61] Waterfield MD, Scrace GT, Whittle N, Stroobant P, Johnsson A, Wasteson Å, Westermark B, Heldin C-H, San Huang J, Deuel TF (1983). Platelet-derived growth factor is structurally related to the putative transforming protein p28 sis of simian sarcoma virus. Nature.

[CR62] Weller ML, Amornphimoltham P, Schmidt M, Wilson PA, Gutkind JS, Chiorini JA (2010). Epidermal growth factor receptor is a co-receptor for adeno-associated virus serotype 6. Nat Med.

[CR63] Wilson EB, El-Jawhari JJ, Neilson AL, Hall GD, Melcher AA, Meade JL, Cook GP (2011). Human tumour immune evasion via TGF-β blocks NK cell activation but not survival allowing therapeutic restoration of anti-tumour activity. PLoS ONE.

[CR64] Wise LM, Ueda N, Dryden NH, Fleming SB, Caesar C, Roufail S, Achen MG, Stacker SA, Mercer AA (2003). Viral vascular endothelial growth factors vary extensively in amino acid sequence, receptor-binding specificities, and the ability to induce vascular permeability yet are uniformly active mitogens. J Biol Chem.

[CR65] Witsch E, Sela M, Yarden YJP (2010) Roles for growth factors in cancer progression. Physiology 10.1152/physiol.00045.2009 PMC3062054 20430953

[CR66] Wu K, Oberstein A, Wang W, Shenk TJ (2018). Role of PDGF receptor-α during human cytomegalovirus entry into fibroblasts. Proc Natl Acad Sci.

[CR67] Xu Y, Shi Y, Yuan Q, Liu X, Yan B, Chen L, Tao Y, Cao YJ (2013). Epstein-Barr Virus encoded LMP1 regulates cyclin D1 promoter activity by nuclear EGFR and STAT3 in CNE1 cells. J Exp Clin Cancer Res.

[CR68] Yan Y, Cui H, Guo C, Li J, Huang X, Wei J, Qin QJ (2013). An insulin-like growth factor homologue of Singapore grouper iridovirus modulates cell proliferation, apoptosis and enhances viral replication. J General Virol.

[CR69] Yoo Y, Ueda H, Park K, Flanders KC, Lee YI, Jay G, Kim S-JJ (1996). Regulation of transforming growth factor-beta 1 expression by the hepatitis B virus (HBV) X transactivator. Role in HBV pathogenesis. J Clin Investig.

[CR70] Zhang W, Liu HT (2002). MAPK signal pathways in the regulation of cell proliferation in mammalian cells. Cell Res.

